# Metformin blunts muscle hypertrophy in response to progressive resistance exercise training in older adults: A randomized, double‐blind, placebo‐controlled, multicenter trial: The MASTERS trial

**DOI:** 10.1111/acel.13039

**Published:** 2019-09-26

**Authors:** R. Grace Walton, Cory M. Dungan, Douglas E. Long, S. Craig Tuggle, Kate Kosmac, Bailey D. Peck, Heather M. Bush, Alejandro G. Villasante Tezanos, Gerald McGwin, Samuel T. Windham, Fernando Ovalle, Marcas M. Bamman, Philip A. Kern, Charlotte A. Peterson

**Affiliations:** ^1^ Center for Muscle Biology College of Health Sciences University of Kentucky Lexington Kentucky; ^2^ UAB Center for Exercise Medicine University of Alabama at Birmingham Birmingham Alabama; ^3^ Department of Biostatistics College of Public Health University of Kentucky Lexington Kentucky; ^4^ Department of Statistics College of Arts & Sciences University of Kentucky Lexington Kentucky; ^5^ Department of Epidemiology School of Public Health University of Alabama at Birmingham Birmingham Alabama; ^6^ Department of Surgery School of Medicine University of Alabama at Birmingham Birmingham Alabama; ^7^ Department of Medicine University of Alabama at Birmingham Birmingham Alabama; ^8^ Department of Cell, Developmental & Integrative Biology School of Medicine University of Alabama at Birmingham Birmingham Alabama; ^9^ Division of Endocrinology Department of Medicine University of Kentucky Lexington Kentucky

**Keywords:** aging, exercise–drug interaction, muscle mass, strength training

## Abstract

Progressive resistance exercise training (PRT) is the most effective known intervention for combating aging skeletal muscle atrophy. However, the hypertrophic response to PRT is variable, and this may be due to muscle inflammation susceptibility. Metformin reduces inflammation, so we hypothesized that metformin would augment the muscle response to PRT in healthy women and men aged 65 and older. In a randomized, double‐blind trial, participants received 1,700 mg/day metformin (*N* = 46) or placebo (*N* = 48) throughout the study, and all subjects performed 14 weeks of supervised PRT. Although responses to PRT varied, placebo gained more lean body mass (*p* = .003) and thigh muscle mass (*p* < .001) than metformin. CT scan showed that increases in thigh muscle area (*p* = .005) and density (*p* = .020) were greater in placebo versus metformin. There was a trend for blunted strength gains in metformin that did not reach statistical significance. Analyses of vastus lateralis muscle biopsies showed that metformin did not affect fiber hypertrophy, or increases in satellite cell or macrophage abundance with PRT. However, placebo had decreased type I fiber percentage while metformin did not (*p* = .007). Metformin led to an increase in AMPK signaling, and a trend for blunted increases in mTORC1 signaling in response to PRT. These results underscore the benefits of PRT in older adults, but metformin negatively impacts the hypertrophic response to resistance training in healthy older individuals. ClinicalTrials.gov Identifier: NCT02308228.

## 1 INTRODUCTION

In elderly persons, muscle mass is highly correlated with limited mobility, disability, and mortality (Han, Bokshan, Marcaccio, DePasse, & Daniels, 2018). Resistance exercise training (RT) is the most effective therapy for sarcopenia and has been shown to increase muscle fiber size, muscle mass, and strength (Taaffe, Pruitt, Pyka, Guido, & Marcus, 1996). Accordingly, RT has also been shown to improve activities of daily living, overall health, and quality of life in elderly individuals (Hunter, McCarthy, & Bamman, 2004). However, hypertrophic and functional improvements following RT vary among individuals, with some people completely failing to experience muscle hypertrophy (Stec et al., 2017). Numerous conditions may contribute to the nonresponder phenotype in some older adults, including anabolic resistance (Fry & Rasmussen, 2011), chronic low‐grade inflammation (Dalle, Rossmeislova, & Koppo, 2017), and muscle‐specific inflammation (Merritt et al., 2013).

Metformin has been among the top 10 most widely prescribed drugs in the United States for nearly two decades (ClinCalc DrugStats Database, clincalc.com/DrugStats; Marshall, 2017). Metformin enhances insulin‐stimulated glucose uptake in rodents (Peixoto et al., 2017) and in muscle cell culture (Galuska, Nolte, Zierath, & Wallberg‐Henriksson, 1994), although in humans its main effect is to inhibit hepatic glucose output (Yu, Kruszynska, Mulford, & Olefsky, 1999). Metformin has also been shown to reduce inflammation in muscle (Amin, Hussein, Yassa, Hassan, & Rashed, 2018; Peixoto et al., 2017). At the tissue level, macrophages are important mediators of inflammatory signaling; metformin promotes polarization of pro‐inflammatory M1 macrophages to anti‐inflammatory M2 macrophages in murine adipose tissue (Amin et al., 2018) and bone marrow‐derived monocytes (Cameron et al., 2016). We recently showed that resident M2 muscle macrophages are associated with exercise‐mediated increases in skeletal muscle satellite cells and fiber size (Walton et al., 2019). In addition, we previously found that resting muscle tissue of many older adults is in a heightened, local state of inflammation susceptibility (Merritt et al., 2013). Based on these combined data, we hypothesized that metformin may improve the muscle response to RT by increasing the abundance of M2 macrophages, thereby reducing muscle inflammation.

In the absence of RT, metformin appears to delay lean mass loss in men with type 2 diabetes (Lee et al., 2011). However, when adults with prediabetes underwent concurrent aerobic and resistance training, metformin appeared to blunt exercise‐induced gains in lean mass (Malin, Gerber, Chipkin, & Braun, 2012). It is unknown whether metformin treatment can augment lean mass gains during RT in healthy older individuals. In The Metformin to Augment Strength Training Effective Response in Seniors (MASTERS) randomized trial, we determined whether the hypertrophic response to progressive RT (PRT) would be improved by the addition of metformin compared to placebo (Long et al., 2017).

## 2 RESULTS

### 2.1 Study participants and design

Baseline characteristics of study participants are given in Table 1. At the University of Alabama at Birmingham (UAB), 104 subjects were screened and 76 were randomized. At the University of Kentucky (UK), 41 subjects were screened and 33 were randomized. Of 109 randomized subjects, 15 participants discontinued the study (*n* = 6 due to adverse events), resulting in a final analytic sample of 94 (86%) who completed the study (*n* = 46 Metformin; *n* = 48 Placebo). Baseline characteristics and dropout rates were similar between treatment groups. The Consort diagram is shown in Appendix S1: Figuer 1. Among randomized subjects, 56% were female. Median age was 69.3 (interquartile range [IQR] 66.9–73.0), and mean BMI was 26.3 (*SD* 3.2). The participants tended to be high functioning, with a median Short Physical Performance Battery (SPPB) score of 11.0 (IQR 10–12). Similarly, questionnaire‐based indices suggested high function in our participants: Median Physical Activity Scale for the Elderly score was 158.7 (IQR 124.3–207.9); median 36‐Item Short Form Survey Instrument (SF‐36) Physical component norm‐based score was 54.8 (IQR 49.4–57.6); and median SF‐36 Mental component norm‐based score was 56.5 (IQR 53.8–59.2). The 109 randomized subjects included 105 Caucasians, three African Americans, and one Asian.

**Table 1:** Baseline characteristics of study participants

	Placebo	Metformin
Randomized	*N* = 55	*N* = 54
Discontinued	7 (13%)	8 (15%)
Site		
Lexington, KY	17 (31%)	16 (30%)
Birmingham, AL	38 (69%)	38 (70%)
Sex, all randomized participants		
Female	32 (58%)	29 (54%)
Male	23 (42%)	25 (46%)
Sex, participants who completed the study		
Female	29 (60%)	22 (48%)
Male	19 (40%)	24 (52%)
Age median (IQR), range	69.3 (66.8–74.0), 64.4–82.8	69.4 (66.8–72.3), 64.8–91.2
BMI mean (*SD*), range	25.7 (3.1), 18.6–30.3	26.9 (3.1), 20.2–33.9
Function		
Short Physical Performance Battery (SPPB) median (IQR), range	11 (11–12), 7–12	11 (10–12), 5–12
Physical Activity Scale for the Elderly (PASE) median (IQR), range	165.7 (133.4–210.5), 52.5–348.2	155.5 (115.8–207.4), 55.0–492.0
36‐Item Short Form (SF−36) Physical^a^ median (IQR), range	55.2 (49.5–57.6), 35.0–62.4	54.7 (49.1–57.5), 27.9–67.9
36‐Item Short Form (SF−36) Mental^a^ median (IQR), range	56.5 (54.6–59.1), 40.7–63.0	56.7 (52.3–59.3), 17.5–65.5

^a^ Component norm‐based score.

**Figure 1:**
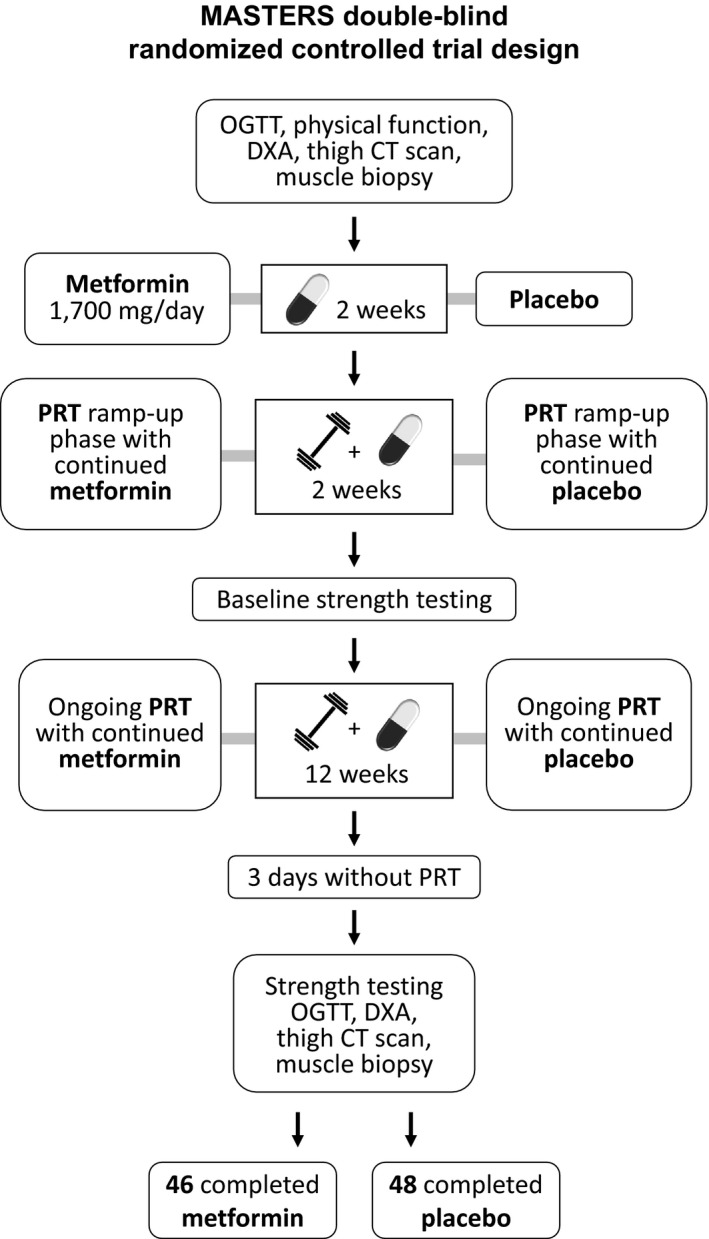
A schematic representation of the study design. CT, computed tomography; DXA, dual‐energy X‐ray absorptiometry; OGTT, oral glucose tolerance test; PRT, progressive resistance training

An overview of the study design is shown in Figure 1. Subjects were randomized to receive either placebo or metformin, which was titrated up to the target dose of 1,700 mg/day, consistent with doses that are prescribed for diabetes and prediabetes (Hess, Unger, Madea, Stratmann, & Tschoepe, 2018), for the duration of the trial. Following a 2‐week drug wash‐in period, and a 2‐week PRT familiarization and ramp‐up period, baseline strength testing was performed. All participants then continued to perform 12 more weeks of supervised, variable intensity, bilateral, upper, and lower body PRT. Participants received metformin or placebo for the entire duration of PRT and through post‐training assessments. Dual‐energy X‐ray absorptiometry (DXA) scans, mid‐thigh computed tomography (CT) scans, muscle biopsies, and oral glucose tolerance tests were performed at baseline and 3 days after the final exercise bout, to assess chronic effects of the intervention. While 60% of participants who completed the placebo arm were female, 48% of participants who completed the metformin arm were female. Although sex distribution was not significantly different between groups (*χ* ^2^ (*df* = 1) = 1.50, *p* = .221), the sex distribution led to significant differences in baseline measures. To account for sex differences at baseline and following training, we compared percent changes in all measured outcome variables except for those that were already percent‐based (percent fat and percent fiber type frequency).

Medication and exercise compliance data are given in Appendix S1. Adverse events were more likely to occur with metformin (*χ* ^2^ (*df* = 1) = 9.82, *p* = .002) (Appendix S1: Table 1). Deviations from the study protocol included randomization of 13 obese subjects (BMI 30–33.9). Additionally, five subjects completed fewer than 37 exercise sessions prior to undergoing the final muscle biopsy. A summary and explanation of missing data are given in Appendix S1: Table 2.

Nearly all participants underwent some beneficial physiological adaptations in response to PRT. Appendix S2: Table 1 shows the effect of PRT for all measured outcomes in those who completed the trial. Appendix S2: Table 2 shows the effects of PRT within each treatment group.

### 2.2 Metformin inhibits PRT‐induced gains in total lean mass and thigh muscle mass; metformin trends toward inhibiting strength gains

Changes in body weight, diet, and glucose metabolism are shown in Appendix S3. PRT induced weight loss in most participants, with no effect of metformin. Dietary intake was not affected by PRT, with or without metformin (Appendix S3: Table 1). Although fasting glucose was decreased in placebo by −3.35% (*SD* 7.46) (*p* = .003), metformin had a mean nonsignificant decrease of −1.59% (*SD* 7.99) (*p* = .184); nonetheless, changes in fasting glucose did not differ between treatment groups (*p* = .272) (Appendix S2: Table 2; Appendix S3: Table 1). Insulin sensitivity was improved by PRT in both groups, with no significant difference between groups (Appendix S2: Table 2; Appendix S3: Table 1).

**Figure 2:**
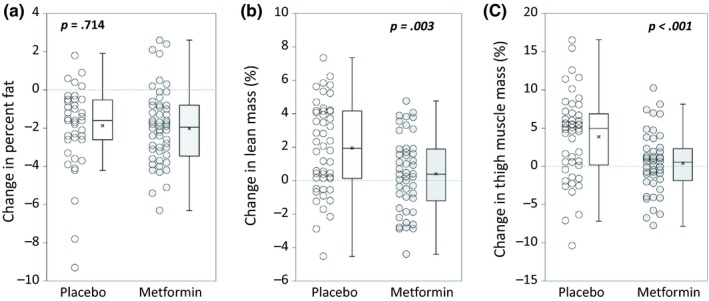
Metformin does not affect decreases in percent fat, but blunts gains in lean mass and thigh muscle mass following PRT. Body composition and bilateral thigh muscle mass were measured using DXA. *N* = 48 placebo, 46 metformin. (a) With PRT, percent fat was reduced in most participants, and this reduction was not affected by metformin (*p* = .714). (b) Percent change in lean mass was larger in placebo than in metformin (*p* = .003), and (c) percent change in thigh muscle mass was also larger in placebo than in metformin (*p* < .001). Student's *t* test. Box plots indicate the mean (×) and median (‐ ‐ ‐ ‐ ‐ ‐ ‐ ‐), and whiskers indicate the upper and lower quartiles

We used DXA to assess body composition at baseline and after PRT (Figure 2). PRT led to a reduction in percent fat, with no difference between groups; mean decrease in percent fat was −1.86 (*SD* 2.08) with placebo and −2.02 (*SD* 1.95) with metformin (between groups *p* = .714) (Figure 2a). However, metformin prevented gains in lean mass with PRT (between groups *p* = .003); placebo gained 1.95% (*SD* 2.69) lean mass (*p* < .001), while the 0.41% change (*SD* 2.25) in metformin did not reach significance (*p* = .218) (Figure 2b; Appendix S2: Table 2). Metformin also blocked thigh muscle mass gains (between groups *p* < .001), with placebo gaining 3.90% (*SD* 5.54) (*p* < .001), and metformin showing no significant gain (0.45%, *SD* 3.95) (*p* = .441) (Figure 2c; Appendix S2: Table 2).

With PRT, increases in strength tended to be lower with metformin, but differences were not statistically significant (Table 2). Knee extension 1 repetition maximum (RM) increased 23.1% (*SD* 18.9) in placebo and 15.3% (*SD* 18.5) in metformin (between groups *p* = .055); knee extension isometric strength increased 11.8% (*SD* 12.7) with placebo compared to 6.7% (*SD* 14.5) with metformin (between groups *p* = .082); and peak knee extension power increased 29.4% (*SD* 40.7) in placebo versus 14.3% (*SD* 35.7) in metformin (between groups *p* = .064). Relative strength (knee extension kg/bilateral thigh muscle mass kg) gains following PRT were similar in both groups, with placebo improving 19.7% (*SD* 19.0) and metformin improving 14.5% (*SD* 17.9) (between groups *p* = .188).

**Table 2:** Changes in strength and thigh muscle CT following 14 weeks of PRT

Outcome measure	Placebo				Metformin				*p* =
	*N*	Baseline	14 week PRT	% change mean (*SD*)	*N*	Baseline	14 week PRT	% change mean (*SD*)	Effect of drug
Strength testing^a^									
Knee extension 1 RM (kg) mean (*SD*)	45	39.8 (18.8)	48.6 (22.5)	23.1 (18.9)	44	53.4^b^ (22.6)	60.6 (24.4)	15.3 (18.5)	0.055
Maximum voluntary isometric contraction (Nm) mean (*SD*)	46	137.3 (41.5)	152.3 (45.3)	11.8 (12.7)	45	168.0^b^ (53.3)	177.0 (56.1)	6.7 (14.5)	0.082
Power (W) mean (*SD*)	46	256.7 (120.3)	310.0 (130.3)	29.4 (40.7)	44	327.7^b^ (130.0)	368.8 (140.1)	14.3 (35.7)	0.064
Relative strength (kg/kg)^c^ mean (*SD*)	45	3.43 (3.27)	3.99 (3.70)	19.7 (19.0)	44	4.40 (4.49)	4.72 (4.70)	14.5 (17.9)	0.188
Thigh muscle CT^d^									
Low‐density muscle area, 0–34 HU (cm^2^) mean (*SD*)	40	23.2 (7.3)	21.5 (6.8)	−6.74 (11.6)	37	26.9^b^ (9.6)	25.6 (8.7)	−3.86 (10.6)	0.257
Normal density muscle area, 35–100 HU (cm^2^) mean (*SD*)	40	78.8 (20.5)	86.9 (22.2)	10.5 (7.0)	37	97.2^b^ (26.7)	101.5 (29.4)	4.16 (9.1)	0.001

Note

PRT, progressive resistance training; *SD*, standard deviation.

a Baseline strength testing was performed at week 2 of PRT, RM = repetition maximum.

b treatment groups were significantly different at baseline.

c kg knee extension/kg thigh muscle mass, CT = computed tomography.

d CT analyses are based on the mean of both legs, HU = Hounsfield Units.

### 2.3 Metformin did not affect vastus lateralis fiber hypertrophy; metformin inhibited decreases in type I fiber frequency

**Figure 3:**
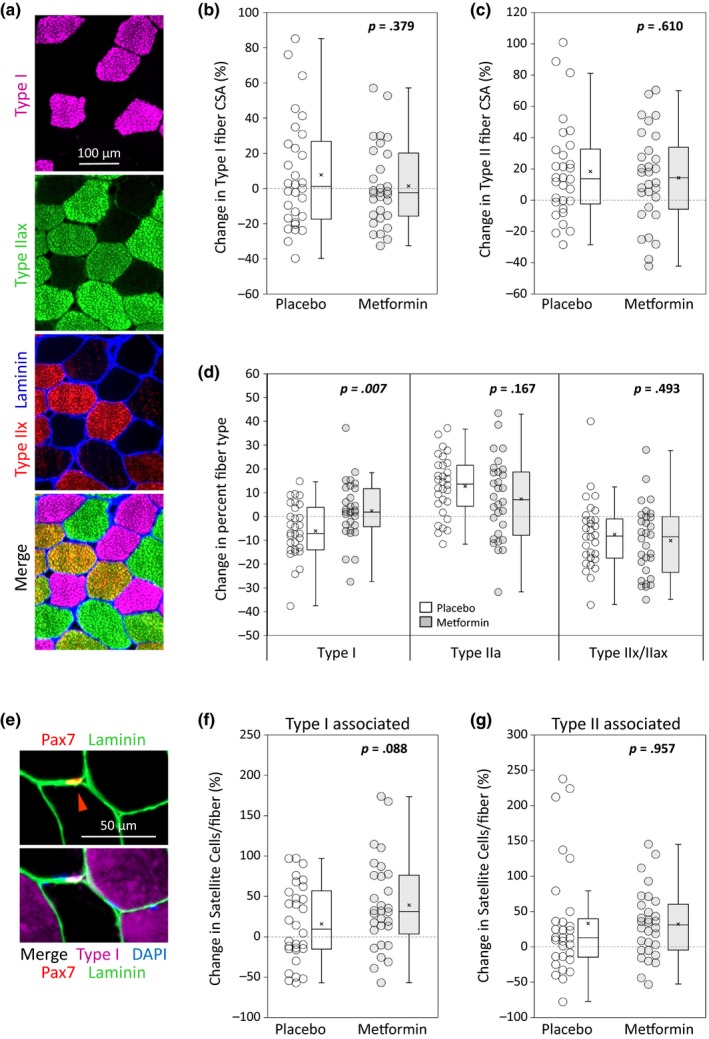
With PRT, metformin does not affect fiber hypertrophy, but inhibits increased type I fiber frequency, without significantly affecting satellite cell expansion. Immunohistochemistry on pre‐ and post‐PRT biopsies was performed in 30 subjects/group. (a) Representative immunoflourescent fiber typing, showing staining of type I myosin heavy chain (purple), type IIax myosin heavy chain (green), type IIx myosin heavy chain (red), and laminin for fiber borders (blue). Type I/II hybrid fibers were excluded from analyses. (b) Percent change in type I fiber CSA was negligible and was not affected by metformin (*p* = .379). (c) PRT caused type II fiber hypertrophy in most subjects, and percent change in type II fiber CSA was not affected by metformin (*p* = .610). (d) Changes in fiber type frequency (percent fiber type): While placebo had reduced type I fiber frequency following PRT, metformin blunted this adaptation (*p* = .007); however, metformin did not affect increases in type IIa fiber frequency (*p* = .167), or decreases in type IIx/IIax fiber frequency (*p* = .493). (e) Representative immunoflourescent stain for fiber type‐specific satellite cells, using Pax7 to identify satellite cells (red), laminin for fiber borders (green), type I myosin heavy chain (purple), and DAPI for nuclei (blue). The red carrot indicates a type II fiber‐associated satellite cell. (f) Compared to placebo, metformin had an increased mean, but not significant, percent change in type I‐associated satellite cells/type I fibers (*p* = .088). (g) The percent change in type II‐associated satellite cells/type II fibers was not affected by metformin. Student's *t* test. Box plots indicate the mean (×) and median (‐ ‐ ‐ ‐ ‐ ‐ ‐ ‐), and whiskers indicate the upper and lower quartiles

Fiber cross‐sectional area (CSA) was determined by immunohistochemistry for type I fibers, type II fibers, and fiber borders (laminin) in vastus lateralis (VL) biopsies (Figure 3a). Metformin did not affect type I fiber CSA with PRT; mean change in type I CSA was 7.64% (*SD* 31.6) in placebo and 1.30% (*SD* 23.1) in metformin (between groups *p* = .379) (Figure 3b). Metformin also did not affect type II fiber hypertrophy with PRT; mean type II CSA change in placebo was 18.5% (*SD* 31.5) versus 14.5% (*SD* 29.7) in metformin (between groups *p* = .610) (Figure 3c). Since metformin inhibited lean mass gains measured by DXA, we assessed whether changes in type II fiber CSA accurately reflect changes in bilateral thigh muscle mass (by DXA). There was a trend toward a positive correlation between changes in type II fiber CSA and thigh muscle mass (*R* ^2^ = 0.071, *p* = .058) (Appendix S4: Figure 1a).

Metformin inhibited shifts in fiber type frequency following PRT. Metformin prevented decreased type I fiber frequency with PRT (between groups *p* = .007); placebo decreased type I fiber frequency by 6.06% (*SD* 11.5) (*p* = .007), while metformin had a nonsignificant increase of 2.50% (*SD* 12.4) (*p* = .278) (Figure 3d; Appendix S2: Table 2). Both groups gained type IIa fiber frequency, with placebo increasing by 12.9% (*SD* 12.6), and metformin increasing by 7.48% (*SD* 17.0) (between groups *p* = .167) (Figure 3d). Both groups also had similar decreases in type IIx/IIax fiber frequency following PRT, with placebo decreasing by −7.49% (*SD* 14.4) and metformin decreasing by −10.1% (*SD* 15.0) (between groups *p* = .493) (Figure 3d).

### 2.4 Metformin did not affect increases in satellite cells

Our group has previously shown that satellite cell (muscle stem cell) accretion is associated with fiber hypertrophy (Fry et al., 2014; Petrella, Kim, Mayhew, Cross, & Bamman, 2008). We therefore determined whether metformin would affect fiber type‐specific satellite cell content using immunohistochemistry for Pax7, type I fibers, and fiber borders (laminin) (Figure 3e). Following PRT, there was a trend toward greater increases in type I‐associated satellite cells with metformin. Placebo increased type I‐associated satellite cells/type I fiber by 16.1% (*SD* 48.0), while metformin increased type I‐associated satellite cells/type I fiber by 39.4% (*SD* 56.0) (between groups *p* = .088) (Figure 3f). Type II‐associated satellite cell accretion did not differ between groups, with placebo increasing by 33.0% (*SD* 79.1) and metformin increasing by 32.1% (*SD* 47.8) (between groups *p* = .957) (Figure 3g).

### 2.5 Metformin inhibits PRT‐induced increases in thigh muscle cross‐sectional area and density

**Figure 4:**
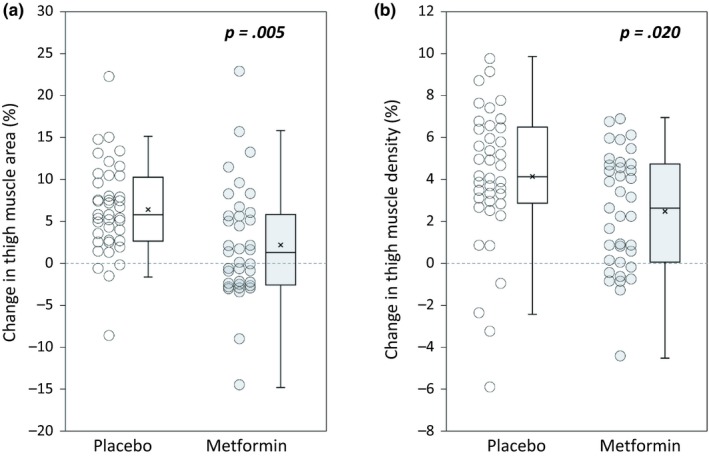
Metformin blunts increased thigh muscle area and density resulting from PRT. Using CT, we assessed thigh muscle cross‐sectional area (cm^2^) and density (Hounsfield Units, HU), using the average of both legs. *N* = 40 placebo, 37 metformin. (a) The percent change in thigh muscle area was significantly larger in placebo versus metformin (*p* = .005), and (b) placebo also had a larger percent change in thigh muscle density (*p* = .020). Student's *t* test. Box plots indicate the mean (×) and median (‐ ‐ ‐ ‐ ‐ ‐ ‐ ‐), and whiskers indicate the upper and lower quartiles

Metformin blocked PRT‐induced gains in lean mass by DXA. Thus, we determined whether increases in thigh muscle CSA and density, assessed by mid‐thigh CT scans, would be affected by metformin treatment (Figure 4). Decreases in low‐density muscle, which contains more intramyocellular lipid than normal density muscle, were similar between groups, with placebo losing −6.74% (*SD* 11.6), and metformin losing −3.86% (*SD* 10.6) (between groups *p* = .257) (Table 2). However, placebo gained significantly more normal density muscle area than metformin, with a mean gain of 10.5% (*SD* 7.01) with placebo versus 4.16% (*SD* 9.11) with metformin (between groups *p* = .001) (Table 2). Since the thigh muscles contain more normal than low‐density muscle, placebo gained significantly more total muscle area than metformin. Mean gain in total muscle area was 6.43% (*SD* 5.45) with placebo, versus 2.27% (*SD* 6.91) with metformin (between groups *p* = .005) (Figure 4a). With resistance training, mean changes in type II fiber CSA and CT thigh muscle area have been shown to be comparable (Nilwik et al., 2013). Thus, we assessed the accuracy of these measures in our cohort and found a significant positive correlation between mean changes in type II fiber CSA and CT thigh muscle area (*R* ^2^ = 0.116, *p* = .014) (Appendix S4, Figure 1b). However, changes in thigh muscle mass (by DXA) and changes in total thigh muscle area (by CT) were more tightly associated with each other (*R* ^2^ = 0.325, *p* < .0001) (Appendix S4, Figure 1c) than with changes in type II fiber CSA. Consistent with lower gains in normal density muscle area, metformin displayed a significantly smaller increase in average muscle density; placebo increased muscle density by 4.13% (*SD* 3.27), while metformin increased muscle density by 2.49% (*SD* 2.75) (between groups *p* = .020) (Figure 4b).

### 2.6 Metformin does not affect muscle macrophage increases with PRT

**Figure 5:**
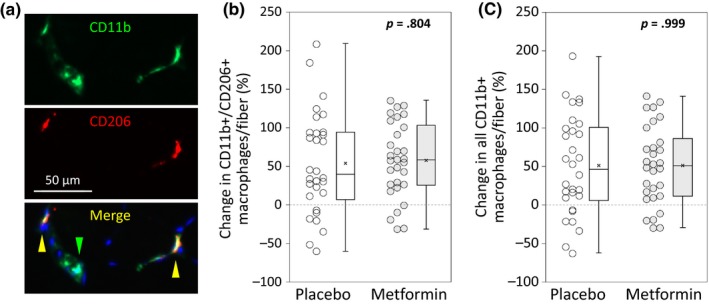
Metformin does not affect increases in skeletal muscle macrophages with PRT. Immunohistochemistry on pre‐ and post‐PRT biopsies was performed in 30 subjects/group. (a) Representative immunoflourescent macrophage identification, showing staining for CD11b (green), CD206 (red), and DAPI for nuclei (blue). The green carrot indicates a CD11b + macrophage, and the yellow carrots indicate CD11b+/CD206 + macrophages. When given with PRT, metformin did not affect the percent change in (b) CD11b+/CD206 + macrophages per fiber (*p* = .804), or in (c) all CD11b + macrophages per fiber (0.999). Student's *t* test. Box plots indicate the mean (×) and median (‐ ‐ ‐ ‐ ‐ ‐ ‐ ‐), and whiskers indicate the upper and lower quartiles

We originally hypothesized that metformin would enhance skeletal muscle hypertrophy by increasing resident M2 macrophages. We therefore performed immunohistochemistry for anti‐inflammatory M2 macrophages (CD11b+/CD206+) and total macrophages (CD11b+) in VL biopsies obtained before and after the intervention (Figure 5a). Although both cell populations increased following PRT, neither was affected by metformin. CD11b+/CD206 + macrophages increased by 53.6% (*SD* 66.3) in placebo and by 57.3% (*SD* 48.6) in metformin (between groups *p* = .804) (Figure 5b). Total macrophages increased by 50.9% (*SD* 64.6) with placebo and by 51.0% (*SD* 50.6) with metformin (between groups *p* = .999) (Figure 5c).

### 2.7 Chronic metformin administration increases basal AMPK and ACC phosphorylation while blunting RPS6 activation following PRT

**Figure 6:**
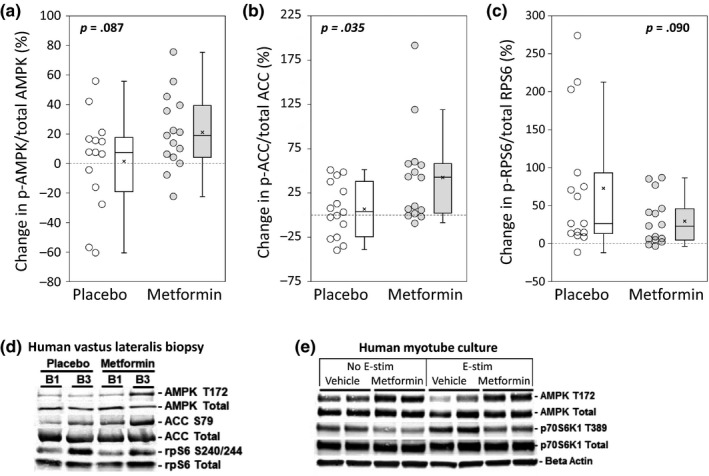
Metformin administration with PRT increases mean basal AMPK and ACC phosphorylation, and blunts mean RPS6 activation following PRT. Western blotting was performed on baseline and post‐PRT vastus lateralis biopsies from 15 subjects/group. One placebo outlier was removed from AMPK analysis. (a) Following PRT, metformin had a higher percent change in basal phospho‐AMPK:total AMPK ratio that did not reach statistical significance (*p* = .087). (b) The metformin group also had a significantly higher percent change in basal phospho‐ACC:total ACC ratio following PRT (*p* = .035). (c) The placebo group had a greater mean increase in basal phospho‐RPS6:total RPS6 ratio that did not reach statistical significance (*p* = .090). (d) Representative Western blots of human muscle biopsies. (e) In cultured human myotubes, treatment with metformin blunts phosphorylation of p70S6K1 following electrical stimulation. Student's *t* test. Box plots indicate the mean (×) and median (‐ ‐ ‐ ‐ ‐ ‐ ‐ ‐), and whiskers indicate the upper and lower quartiles

Acute resistance exercise transiently activates AMPK; however, metformin inhibits mitochondrial complex I, causing decreased cellular energy availability, and ultimately leading to chronic AMPK activation (Musi et al., 2002). In order to determine whether metformin affects chronic AMPK‐mediated signaling in the context of PRT, we used Western blots to quantify the phosphorylation of AMPK, its downstream target ACC, and RPS6, a downstream target of mTORC1 in VL biopsies from 15 subjects/group. With PRT, the mean ratio of phospho‐AMPK:total AMPK was increased by 1.6% (*SD* 8.8) in placebo and by 21.3% (*SD* 6.6) in metformin (between groups *p* = .087) (Figure 6a). Consistent with chronic AMPK activation, metformin had a significantly larger increase in ACC phosphorylation. Phospho‐ACC increased by 6.2% (*SD* 7.9) with placebo, versus 42.2% (*SD* 13.9) with metformin (between groups *p* = .035) (Figure 6b). Following PRT, RPS6 phosphorylation increased by 73.1% (*SD* 22.6) in placebo and by 29.9% (*SD* 8.2) in metformin (between groups *p* = .090) (Figure 6c,d).

We next modeled exercise in primary human myotubes in order to determine whether metformin affects AMPK and mTORC1 signaling in response to an anabolic exercise stimulus. Human myogenic progenitor cells were differentiated into myotubes and treated with metformin during 8 hr of high‐frequency electrical pulse stimulation (Tarum, Folkesson, Atherton, & Kadi, 2017). Western blots were used to quantify phospho‐AMPK:total AMPK and phospho‐p70S6K1:total p70S6K1 (an immediate target of mTOR). Consistent with chronic changes in VL biopsies, metformin induced greater phospho‐AMPK in cultured myotubes, irrespective of whether they received electrical stimulation. Following electrical stimulation, metformin inhibited mean p70S6K1 phosphorylation (Figure 6e).

## 3 DISCUSSION

In men and women aged 65 and older, 14 weeks of PRT induced the expected increases in muscle mass and strength. However, metformin administered along with PRT inhibited these gains. DXA showed that metformin gained significantly less total lean mass and less thigh muscle mass than placebo. Likewise, CT analysis indicated that normal density thigh muscle area increased following PRT, but metformin blunted this gain. Our observations are consistent with Malin et al. (2012), who reported that metformin inhibits gains in fat‐free mass in response to concurrent aerobic and resistance training in subjects with prediabetes.

Metformin may inhibit muscle hypertrophy via inhibition of mTORC1, leading to decreased muscle protein synthesis or increased autophagy. Metformin indirectly activates AMPK, a major mTORC1 inhibitory kinase, leading to reduced hypertrophy in muscle cell culture and rodents (Kwon & Querfurth, 2015; Lantier et al., 2010). In human muscle, metformin reduces the expression of mTORC1‐related genes in older individuals with impaired glucose tolerance (Kulkarni et al., 2018). In keeping with these data, we observed lower mean RPS6 activation in participants randomized to metformin and inhibition of p70S6K1 phosphorylation in cultured myotubes following metformin treatment during electrical pulse stimulation. Thus, acute and/or chronic blunting of mTORC1 activation may contribute to the decreased lean mass gains in metformin‐treated subjects.

Strength and power also increased in response to PRT, but we found trends for blunted gains with metformin treatment. Boule et al. (2013) reported that following 22 weeks of resistance training in middle‐aged subjects with type 2 diabetes, metformin inhibited muscle mass gains without affecting strength. Since our study neither confirms nor contradicts Boule et al., additional studies are warranted to determine how metformin affects strength.

Although muscle mass gains with PRT were lower with metformin, it did not affect changes in muscle fiber CSA. One study reported that following resistance training, increased type II fiber CSA drives increases in thigh muscle area (by CT), but this paper reported mean changes for the entire cohort rather than correlations between the techniques (Nilwik et al., 2013). In another report, VL volume was not correlated with increased fiber CSA in young men following resistance training (Mobley et al., 2018). Compared to measures of whole muscle size, measures of fiber CSA are probably more vulnerable to sampling and technical error, causing increased variance and decreasing statistical power. This idea is supported by our supplemental data showing that CT and DXA measures are highly correlated with each other, while fiber CSA measures are not as highly correlated with CT or DXA. These data suggest that CT and DXA may be more reliable indicators of muscle hypertrophy than fiber CSA. Metformin also did not affect decreases in type IIx/IIax fiber frequency, nor gains in satellite cells or resident macrophage abundance. It is therefore unlikely that changes in these parameters contribute to altered PRT responses with metformin.

Metformin blunted reductions in the frequency of type I fibers, which typically contain more lipid than type II fibers (Schrauwen‐Hinderling, Hesselink, Schrauwen, & Kooi, 2006). Furthermore, placebo had significantly larger improvements in muscle density, assessed by CT. Since lower tissue density is associated with a more lipid‐rich environment, these findings suggest a greater decrease in muscle lipid content in placebo following PRT. Metformin inhibits mitochondrial complex I (Fontaine, 2018), which could lead to inhibition of fatty acid oxidation and increased intramyocellular lipid. Although studies in sedentary humans have shown either no effect (Rasouli et al., 2005) or decreased (Teranishi et al., 2007) intramyocellular lipid following metformin treatment, the increased metabolic demands during PRT may require increased mitochondrial complex I activity, and metformin may interfere with this process. Considering that we also observed increased phosphorylation of AMPK and ACC (which should promote lipid oxidation) in metformin following PRT, we suspect that metformin‐mediated inhibition of complex I counteracts and overrides the effects of AMPK activation, leading to decreased lipid oxidation. Consistent with this idea, metformin was recently shown to abrogate aerobic exercise training‐induced increases in skeletal muscle mitochondrial respiration in older adults (Konopka et al., 2018).

Other outcome measures included body weight, diet, and glucose metabolism. Both groups lost weight following 14 weeks of PRT, and metformin did not affect weight loss. Furthermore, neither PRT nor metformin affected total daily caloric or protein intake, indicating that our observations cannot be explained by dietary changes. Although the metformin‐treated subjects reported more GI side effects, this did not result in discontinuation of drug and did not affect exercise adherence in our generally healthy senior population. Metformin also did not affect changes in fasting glucose, glucose tolerance, or insulin sensitivity, all of which improved in response to PRT. It is noteworthy that metformin inhibits improved insulin sensitivity with endurance training (Konopka et al., 2018), indicating that resistance and endurance training may improve insulin sensitivity through differing mechanisms.

One limitation of our trial is that it did not include sedentary control groups. Therefore, we are unable to draw conclusions regarding the effects of metformin alone on muscle mass and strength in generally healthy older adults. The effects of metformin in frail elderly, alone or in combination with resistance exercise, need further study. Although metformin appears to preserve lean mass in sedentary diabetic patients (Lee et al., 2011), it would be important to know whether metformin affects muscle mass and strength gains with PRT in older diabetic patients. Our study was not designed to address these issues, and the majority of our cohort was nonobese and free from metabolic disease.

In conclusion, 14 weeks of progressive resistance training resulted in variable, but significant, gains in total lean mass and thigh muscle mass in healthy adults aged 65 and over. However, these gains were blunted by metformin administration. While relatively few studies have addressed the combined effects of exercise and metformin in humans, all current data indicate that metformin blunts some of the beneficial effects of endurance, resistance, or combined exercise training (reviewed in Konopka & Miller, 2019). Although metformin is highly effective for the prevention and treatment of diabetes, these results do not support the use of metformin to enhance the benefits of physical activity in healthy elderly people.

## 4 EXPERIMENTAL PROCEDURES

### 4.1 Design, setting, and participants

The MASTERS was a randomized, controlled, double‐blind trial comparing the effects of metformin versus placebo during progressive resistance training (PRT) in community‐dwelling seniors. Participants were recruited in Lexington, Kentucky (UK) and Birmingham, Alabama (UAB), and the protocol was approved by both IRBs via IRB Share. Data collection and study interventions occurred at both UK and UAB. The study design is provided in Figure 1 and in our previous publication (Long et al., 2017). The consort diagram is shown in Appendix S1: Figure 1.

Men and women were eligible to participate whether they were aged 65 and over, independently mobile, had a SPPB (Guralnik et al., 1994) score of 4–12, and were nondiabetic. To be included, participants had to have transportation, and to be cognitively intact and capable of providing written informed consent. Appendix S5 provides recruitment methods and exclusion criteria.

### 4.2 Interventions

Subjects were randomized to receive either placebo or metformin for the duration of the trial. Randomization permutation and blinding procedures are described in Appendix S5. Subjects underwent a 2‐week drug or placebo wash‐in period prior to beginning PRT. Those who were randomized to metformin were titrated up to the target dose by taking 1 tablet per day (850 mg) for 7 days, followed by 2 tablets per day (1,700 mg) for the remainder of the trial.

All study subjects underwent 14 weeks of PRT, supervised by trained personnel. In order to train all muscle groups, every workout consisted of the following bilateral, constant load movements: leg press, knee extension, body weight squat progressing to a split squat, calf press, chest press, lat pull down, biceps curl, and triceps press down. We employed a variable intensity prescription across the three training days each week (high/low/high) based on the results of our previous dose–response trial which showed this prescription optimized strength and muscle mass gains in older adults (Stec et al., 2017). Thus, participants performed high‐intensity workouts on Mondays and Fridays; they completed 3 sets of 8–12 repetitions at their 10 repetition maximum (10RM) load, with 60‐ to 90‐s rests between sets. On Wednesdays, resistance loads were reduced ~ 30% and subjects performed rapid concentric contractions with controlled eccentric loading in order to develop explosive power. Continuous progression was incorporated by increasing the resistance load when subjects were able to perform 12 repetitions for 2 of 3 sets on “high” days. Participants were considered compliant with the exercise protocol if they completed 42 ± 5 exercise sessions, including at least three exercise sessions (high/low/high) in a row before follow‐up testing.

All outcome measures, except for strength testing, were performed at baseline, prior to drug initiation, and 3 days after the final bout of training. In order to account for neuromuscular adaptation, baseline strength testing was performed after 2 weeks after PRT initiation.

### 4.3 Body weight, nutrition, and glucose metabolism

We assessed changes in body weight, diet, and glucose metabolism following PRT with placebo or metformin. At baseline and week 16, subjects completed ≥ 3 consecutive days of diet records, which were analyzed with the Nutrition Data System for Research software published by the University of Minnesota (Schakel, 2001). Also at baseline and week 16, standard 2‐hr oral glucose tolerance tests were performed and the Matsuda index (Matsuda & DeFronzo, 1999) was used to calculate insulin sensitivity. The detailed oral glucose tolerance protocol is provided in Appendix S5.

### 4.4 Body composition and strength

Dual‐energy X‐ray absorptiometry scans were performed using a GE Lunar iDXA at baseline and week 16 in order to measure changes in whole‐body lean and fat mass, percent fat, and thigh muscle mass. Scans were analyzed using the GE Lunar software version 10.0.

Leg strength was measured at week 4 (after 2 weeks of PRT) and week 16. Voluntary, dynamic strength was determined using 1 repetition maximum (1RM), defined as the maximal load that a subject can lift at one time, with proper form, through a full range of motion (Petrella, Kim, Tuggle, Hall, & Bamman, 2005). After warming up, subjects performed single repetition trials with increasing resistance until they failed two attempts at a given load. The last load lifted with good form was recorded as the 1RM. Maximum voluntary isometric knee extension strength was measured using a Biodex 4 dynamometer. To control for neuromuscular adaptations that occur in the early phases of resistance training (Moritani & deVries, 1979), week 4 (after 2 weeks of resistance training) was used as the baseline measure for calculating changes in strength. To calculate relative strength, knee extension 1RM was normalized to DXA bilateral thigh muscle mass (Goodpaster et al., 2006). Additional strength testing methods are provided in Appendix S5.

### 4.5 Computed tomography

Computed tomography was used to measure changes in whole thigh muscle size and density. At baseline and week 16, CT images were obtained from the mid‐thigh (defined as the midpoint between the inguinal crease and proximal border of the patella). CT scans were analyzed to delineate thigh muscle and to determine the CSA of low‐density muscle (0–34 Hounsfield Units [HU]), normal density muscle (35–100 HU), and those areas combined (0–100 HU). Mean attenuation (HU) across the mid‐thigh muscle area that included 0–100 HU muscle was also determined. Additional CT methods are given in Appendix S1.

### 4.6 Cell culture

Human myogenic progenitor cells were isolated and passaged as described (Latroche, Weiss‐Gayet, Gitiaux, & Chazaud, 2018) (Appendix S5). Cells were isolated from the VL of two elderly subjects (age >65 years). Cells were differentiated into myotubes for 5 days using MyoCult serum‐free differentiation media (Stemcell Technologies). On the fifth day, fresh media with 10 mM metformin (Sigma‐Aldrich) or vehicle (PBS) was added just prior to electrical pulse stimulation. Cells were then stimulated at 12 V, 1 Hz, 2 ms for 8 hr (EPS, IonOptix C‐Pace EP), followed by immediate protein extraction in RIPA buffer (ThermoFisher) plus Halt protease/phosphatase inhibitor (ThermoFisher) for Western blot analyses as described below.

### 4.7 Immunohistochemistry and western blotting

At baseline and week 16, muscle biopsies were taken from the VL using a 5‐mm Bergstrom needle with suction. A portion of each VL biopsy was mounted in tragacanth gum and frozen for immunohistochemistry on fresh frozen sections. Antibodies against type I, type IIax, and type IIx myosin heavy chain were used to distinguish fiber types, and antilaminin was used to identify fiber borders. Type I/IIa hybrid fibers were excluded from all analyses. Fiber type‐specific satellite cells were identified using antibodies against Pax7, type I myosin heavy chain, and laminin. Macrophages were identified using antibodies against CD11b and CD206. The largest, highest quality pairs of baseline and week 16 mounts were used for quantification of immunohistochemistry (*N* = 30/group). Detailed methods are given in Appendix S5 and in our previous publications (Fry et al., 2014; Kosmac et al., 2018).

In a subset of participant biopsies (*N* = 15/group), we performed Western blots to quantify phospho‐AMPK, total AMPK, phospho‐ACC, total ACC, phospho‐RPS6, and total RPS6, at baseline and 3 days after the final bout of PRT with metformin or placebo. In placebo, one phospho‐AMPK outlier (>3 *SD* above the mean) was excluded from analyses. Detailed methods are given in Appendix S5.

### 4.8 Statistics

Statistical analyses were performed using SAS version 9.4 (SAS Institute). For all outcomes, statistical significance was set at *p* < .05, using two‐sided tests. All continuous measures were summarized with descriptive statistics, and distributions were tested for normality. Normally distributed measures are shown as mean and standard deviation (*SD*). Non‐normally distributed measures are shown as median and IQR. All outcomes were analyzed as observed, and all analyses were performed on the analytic sample (*N* = 94). Paired *t* tests were used to examine changes with PRT. Two‐sample *t* tests were used to determine whether percent change in each outcome measure differed between the metformin and placebo groups; mean estimates with *SD* are presented. The chi‐square test was used to determine whether the distribution of sex or adverse events differed between groups. Pearson's correlation coefficient was used to assess relationships between measures of muscle hypertrophy. Additional statistical methods, including data quality assurance and power calculations, are given in Appendix S5.

## CONFLICT OF INTEREST

The authors have no conflict of interests to disclose.

## AUTHOR CONTRIBUTIONS

CAP, MMB, and PAK designed the study. DEL, SCT, PAK, STW, and FO performed clinical research. CMD, KK, RGW, and BDP carried out immunohistochemistry and Western blotting. RGW, AGVT, HMB, and GM were involved in statistical analyses. RGW, CMD, DEL, CAP, MMB, and PAK interpreted the data and prepared the manuscript. All authors approved the manuscript prior to submission.

## Data Availability

The data that support the findings of this study are available from the corresponding author upon reasonable request.
