# Corrigendum

**DOI:** 10.1111/acel.13098

**Published:** 2020-01-15

Walton, RG, Dungan, CM, Long, DE, et al. Metformin blunts muscle hypertrophy in response to progressive resistance exercise training in older adults: A randomized, double‐blind, placebo‐controlled, multicenter trial: The MASTERS trial. Aging Cell. 2019; 18:e13039. https://doi.org/10.1111/acel.13039

In Table 2, data in row Relative strength (kg/kg)^c^ mean (SD) were incorrect. The correct data is shown as follow:

**Table 2:** Changes in strength and thigh muscle CT following 14 weeks of PRT

Outcome measure	Placebo				Metformin				*p*
	*N*	Baseline	14 week PRT	% change mean (*SD*)	*N*	Baseline	14 week PRT	% change mean (*SD*)	Effect of drug
Strength testing^a^									
Knee extension 1 RM (kg) mean (*SD*)	45	39.8 (18.8)	48.6 (22.5)	23.1 (18.9)	44	53.4^b^ (22.6)	60.6 (24.4)	15.3 (18.5)	0.055
Maximum voluntary isometric contraction (Nm) mean (*SD*)	46	137.3 (41.5)	152.3 (45.3)	11.8 (12.7)	45	168.0^b^ (53.3)	177.0 (56.1)	6.7 (14.5)	0.082
Power (W) mean (*SD*)	46	256.7 (120.3)	310.0 (130.3)	29.4 (40.7)	44	327.7^b^ (130.0)	368.8 (140.1)	14.3 (35.7)	0.064
Relative strength (kg/kg)^c^ mean (*SD*)	44	3.89 (1.48)	4.62 (1.74)	18.9 (19.1)	44	4.60 (1.39)	5.23 (1.58)	14.8 (18.3)	0.30
Thigh muscle CT^d^									
Low‐density muscle area, 0–34 HU (cm^2^) mean (*SD*)	40	23.2 (7.3)	21.5 (6.8)	−6.74 (11.6)	37	26.9^b^ (9.6)	25.6 (8.7)	−3.86 (10.6)	0.257
Normal density muscle area, 35–100 HU (cm^2^) mean (*SD*)	40	78.8 (20.5)	86.9 (22.2)	10.5 (7.0)	37	97.2^b^ (26.7)	101.5 (29.4)	4.16 (9.1)	0.001

Note

PRT, progressive resistance training; *SD*, standard deviation.

a Baseline strength testing was performed at week 2 of PRT, RM = repetition maximum.

b treatment groups were significantly different at baseline.

c kg knee extension/kg thigh muscle mass, CT = computed tomography.

d CT analyses are based on the mean of both legs, HU = Hounsfield Units.

In addition, related measures presented in Appendix S2 under Supporting Information have been updated online.

The authors would like to apologize for the inconvenience caused.

