# Supplementary material for: Metformin blunts muscle hypertrophy in response to progressive resistance exercise training in older adults: A randomized, double‐blind, placebo‐controlled, multicenter trial: The MASTERS trial
Source: Aging Cell. 2019 Sep 26;18(6):e13039. doi: 10.1111/acel.13039 (PMC6826125; doi:10.1111/acel.13039)
Supplement: Supplementary file 1 [file ACEL-18-e13039-s001.docx]

**Appendix S1. Consort diagram, study compliance, adverse events, and missing data summary**

The consort diagram is provided in Figure 1. Study compliance is described below. Adverse events are shown in Table 1; deviations from protocol are shown in Table 2; and missing data are summarized in Table 3.

**
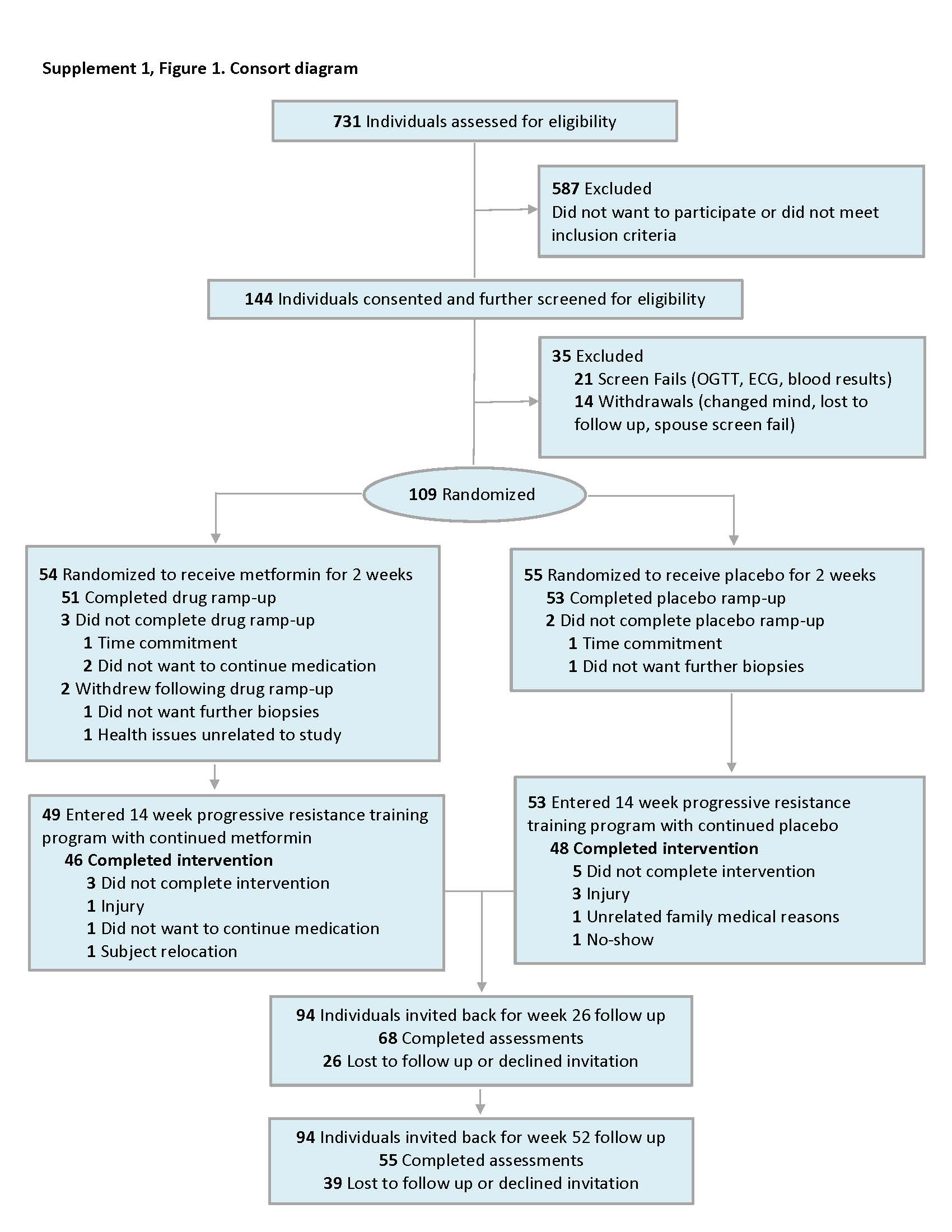
**

**Supplement 1, Figure 1. Consort diagram**

**Compliance.** In those who completed the study, mean number of pills taken was 235 (30.8 SD, range 136-316), yielding a mean daily metformin dose of 1470 mg (176 SD, range 954-1725). In completers, median medication compliance rate was 96.3% (IQR 94.3-98.1%, range 72.7-100%). In completers, the mean number of workouts was 41.4 (2.57 SD, range 33-48), and median workout compliance was 100% (IQR 94.1-100%, range 76-100%). However, 5 subjects completed fewer than 37 exercise sessions prior to undergoing the final muscle biopsy.

Table 1. **Adverse events** in randomized subjects

| **Adverse Event** | **Placebo**  **(Study Related)** | **Placebo**  **(Non-Study Related)** | **Metformin**  **(Study Related)** | **Metformin**  **(Non-Study Related)** | **Total** |
| --- | --- | --- | --- | --- | --- |
| Abscessed tooth* | 0 | 1 | 0 | 0 | **1** |
| Allergic Reaction | 1 | 0 | 0 | 0 | **1** |
| Asthma Attack* | 0 | 0 | 0 | 1 | **1** |
| Back Pain (Upper and Lower regions)* | 2 | 1 | 0 | 1 | **4** |
| Blood Vessel Rupture in Eye* | 0 | 1 | 0 | 0 | **1** |
| Car Wreck* | 0 | 1 | 0 | 1 | **2** |
| Diverticulitis* | 0 | 0 | 0 | 1 | **1** |
| Dizzy, Lightheadedness, Fatigue | 1 | 0 | 1 | 0 | **2** |
| Flu Like Symptoms | 0 | 0 | 1 | 0 | **1** |
| Gastrointestinal Discomfort (Nausea, Diarrhea, Flatulence) | 2 | 0 | 14 | 0 | **16** |
| Hematoma | 1 | 0 | 1 | 0 | **2** |
| Hypotension* | 0 | 0 | 1 | 0 | **1** |
| Knee Pain | 0 | 0 | 1 | 0 | **1** |
| Medication Intolerance | 0 | 0 | 1 | 0 | **1** |
| Muscle Strain | 1 | 0 | 0 | 0 | **1** |
| Shoulder Injury | 0 | 0 | 1 | 0 | **1** |
| Swollen Knee from Spin Class* | 0 | 0 | 0 | 1 | **1** |
| Syncope | 2 | 0 | 0 | 0 | **2** |
| Tender Biopsy Site | 1 | 0 | 1 | 0 | **2** |
| Tendonitis* | 0 | 1 | 0 | 0 | **1** |
| Upset stomach (+ Vomiting) | 0 | 0 | 2 | 0 | **2** |
| Vertigo, Hypotension, and Bradycardia* | 0 | 0 | 1 | 0 | **1** |
| **Total^‡^** | **11** | **5** | **25** | **5** | **46** |

*Unexpected adverse event

**^‡^**Adverse events occurred significantly more often in participants who received metformin (χ-square (DF = 1) = 9.82, p = 0.002).

Table 2. **Explanation** of missing data points

| **Measurement** | Missing |
| --- | --- |
| **CT** |  |
| Based on femur area, CT position was incorrect | 16 |
| For an unknown reason, CT data indicated 45% loss of thigh muscle area, which was not consistent with other data for this subject. | 1 |
| **Strength testing** |  |
| Shoulder pain | 1 |
| Knee pain | 1 |
| Back pain | 1 |
| Test results were not valid because week 4 and week 16 measures were not taken on the same machine. | 2 |
| **Diet** |  |
| Subject failed to complete ≥ 3 days of diet record at baseline or week 16. | 6 |
| **AMPK western blot** |  |
| Data point >3 SD above the mean | 1 |
