# Supplementary material for: Metformin blunts muscle hypertrophy in response to progressive resistance exercise training in older adults: A randomized, double‐blind, placebo‐controlled, multicenter trial: The MASTERS trial
Source: Aging Cell. 2019 Sep 26;18(6):e13039. doi: 10.1111/acel.13039 (PMC6826125; doi:10.1111/acel.13039)
Supplement: Supplementary file 2 [file ACEL-18-e13039-s002.docx]

**Appendix S2. Effects of progressive resistance training (PRT) in all completers (Table 1) and within-group responses (Table 2)**

Table 1. Effects of PRT in all completers

|  | | **All Completers** | | | | | | | | **p =** | |
| --- | --- | --- | --- | --- | --- | --- | --- | --- | --- | --- | --- |
| **OUTCOME MEASURE** | | **N** | | **Baseline** | | **14 week PRT** | | **% change**  mean (SD) | | **Effect of**  **PRT** | |
| **Body weight (kg)**  mean (SD) | | 94 | | 76.1  (13.4) | | 74.6  (13.2) | | -2.0  (3.5) | | *< 0.0001* | |
| **Diet** | | | | | | | | | | | |
| Food intake (kcal/day)  mean (SD) | | 88 | | 1819  (576) | | 1795  (529) | | 2.2  (25.5) | | 0.426 | |
| Protein intake (g/day)  mean (SD) | | 88 | | 75.2  (22.4) | | 71.8  (22.8) | | -0.70  (31.1) | | 0.834 | |
| **Glucose metabolism** | | | | | | | | | | | |
| Fasting glucose (mg/dL)  median (IQR) | | 94 | | 96.0  (88.8-102.0) | | 92.8  (86.8-98.4) | | -2.49  (7.73) | | *0.002* | |
| Insulin Sensitivity Index  (Matsuda), median (IQR) | | 94 | | 4.63  (2.96-6.26) | | 5.19  (3.69-7.62) | | 39.7  (34.2) | | *< 0.0001* | |
| **Body composition (DXA)** | | | | | | | | | | | |
| Percent fat  mean (SD) | | 94 | | 35.0  (7.44) | | 33.1  (7.40) | | -5.53  (6.13) | | *< 0.0001* | |
| Lean mass (kg)  mean (SD) | | 94 | | 46.1  (9.97) | | 46.7  (9.90) | | 1.20  (2.59) | | *< 0.0001* | |
| Bilateral thigh muscle mass (g) mean (SD) | | 94 | | 1074  (248) | | 1095  (249) | | 2.22  (5.11) | | *< 0.001* | |
| **Strength testing**^†^ | | | | | | | | | | | |
| Knee extension 1 RM (kg)  mean (SD) | 89 | | 46.5  (21.8) | | 54.5  (24.1) | | 19.2  (19.0) | | *< 0.0001* | |  |
| Maximum voluntary isometric contraction (Nm)  mean (SD) | 91 | | 152.5  (49.9) | | 164.5  (52.1) | | 9.3  (13.8) | | *< 0.0001* | |  |
| Power (W)  mean (SD) | 90 | | 291.4  (129.5) | | 338.4  (137.6) | | 17.1  (18.5) | | *< 0.0001* | |  |
| Relative strength (kg/kg) ^‡^  mean (SD) | 90 | | 3.91  (3.93) | | 4.35  (4.21) | | 22.0  (38.8) | | *< 0.0001* | |  |

PRT = progressive resistance training, SD = standard deviation, IQR = interquartile range, ^†^Baseline strength testing was performed at week 2 of PRT, RM = repetition maximum, ^‡^kg knee extension/kg thigh muscle mass

Table 1, continued. Effects of PRT in all completers

|  | **All Completers** | | | | | | | | **p =** | | |  |
| --- | --- | --- | --- | --- | --- | --- | --- | --- | --- | --- | --- | --- |
| **OUTCOME MEASURE** | **N** | | **Baseline** | | **14 week PRT** | | **% change**  mean (SD) | | **Effect of**  **PRT** | | |  |
| **Immunohistochemistry** | | | | | | | | | | |  |  |
| Type I fiber CSA (µm^2^)  mean (SD) | 60 | | 4730  (1601) | | 4705  (1260) | | 4.47  (27.6) | | 0.214 | | |  |
| Type II fiber CSA (µm^2^)  mean (SD) | 60 | | 3618  (1383) | | 4113  (1667) | | 16.5  (30.4) | | *< 0.0001* | | |  |
| Type I fiber frequency (%)  mean (SD) | 60 | | 41.6  (13.3) | | 39.8  (12.5) | | -1.78  (12.6) | | 0.278 | | |  |
| Type IIa fiber frequency (%)  mean (SD) | 60 | | 27.2  (13.3) | | 37.4  (15.6) | | 10.2  (15.1) | | *< 0.0001* | | |  |
| Type IIx/IIax fiber frequency (%)  mean (SD) | 60 | | 29.7  (16.5) | | 20.9  (16.3) | | -8.8  (14.6) | | *< 0.0001* | | |  |
| Type I associated satellite cells/Type I fiber, mean (SD) | | 60 | | 0.099  (0.042) | | 0.118  (0.053) | | 27.7  (5.30) | | *< 0.001* | | |
| Type II associated satellite cells/Type II fiber, mean (SD) | | 60 | | 0.067  (0.031) | | 0.083  (0.044) | | 32.5  (0.65) | | *< 0.001* | | |
| CD11b/CD206  macrophages/fiber, mean (SD) | | 60 | | 0.17  (0.10) | | 0.26  (0.17) | | 55.4  (57.7) | | *< 0.001* | | |
| All CD11b  macrophages/fiber, mean (SD) | | 60 | | 0.21  (0.13) | | 0.31  (0.19) | | 50.9  (57.5) | | *< 0.001* | | |
| **Western blotting** | |  | |  | |  | |  | |  | | |
| phospho-AMPK/total AMPK (AU)  mean (SD) | | 29 | | 51.1  (16.4) | | 55.8  (21.3) | | 11.8  (30.5) | | *0.047* | | |
| phospho-ACC1/total ACC1 (AU)  mean (SD) | | 30 | | 19.1  (14.2) | | 22.6  (19.0) | | 24.2  (46.8) | | *0.008* | | |
| phospho-RPS6/total RPS6 (AU)  mean (SD) | | 30 | | 10.7  (7.3) | | 14.7  (10.6) | | 51.5  (68.3) | | *< 0.001* | | |
| **Thigh muscle CT^§^** | |  | |  | |  | |  | |  | | |
| Low density muscle area,  0-34 HU (cm^2^)  mean (SD) | | 77 | | 25.0  (8.60) | | 23.5  (7.97) | | -5.35  (11.1) | | *< 0.0001* | | |
| Normal density muscle area,  35-100 HU (cm^2^)  mean (SD) | | 77 | | 87.7  (25.3) | | 93.9  (26.8) | | 7.44  (8.64) | | *< 0.0001* | | |
| Thigh muscle area, 0-100 HU (cm^2^)  mean (SD) | | 77 | | 112.6  (29.1) | | 117.4  (30.1) | | 4.43  (6.5) | | *< 0.0001* | | |
| Thigh muscle density (HU)  mean (SD) | | 77 | | 46.1  (3.16) | | 47.6  (3.14) | | 3.34  (3.12) | | *< 0.0001* | | |

PRT = progressive resistance training, PRT = progressive resistance training, SD = standard deviation, AU = arbitrary units, CT = computed tomography, **^§^**CT analyses are based on the mean of both legs, HU = Hounsfeild units

Table 2. Within-group responses to PRT

|  | **PLACEBO** | | | **METFORMIN** | | |
| --- | --- | --- | --- | --- | --- | --- |
| **OUTCOME MEASURE** | **N** | **% change**  Mean (SD) | ^†^**p =** | **N** | **% change**  Mean (SD) | ^†^**p =** |
| **Body weight (kg)** | 48 | -1.63 (3.75) | *0.004* | 46 | -2.36 (3.17) | *< 0.0001* |
| **Diet** | | | | | | |
| Food intake (kcal/day) | 45 | -0.56 (22.9) | 0.870 | 43 | 5.0 (27.9) | 0.247 |
| Protein intake (g/day) | 45 | -0.32 (32.3) | 0.947 | 43 | -1.1 (30.2) | 0.812 |
| **Glucose metabolism** | | | | | | |
| Fasting glucose (mg/dL) | 48 | -3.35 (7.46) | *0.003* | 46 | -1.59 (7.99) | 0.184 |
| Insulin Sensitivity Index (Matsuda) | 48 | 36.3 (33.6) | *< 0.0001* | 46 | 43.3 (34.9) | *< 0.0001* |
| **Body composition (DXA)** | | | | | | |
| Percent fat | 48 | -1.86 (2.08) | *< 0.0001* | 46 | -2.02 (1.95) | *< 0.0001* |
| Lean mass (kg) | 48 | 1.95 (2.69) | *< 0.0001* | 46 | 0.41 (2.25) | 0.218 |
| Bilateral thigh muscle mass (g) | 48 | 3.90 (5.54) | *< 0.0001* | 46 | 0.45 (3.95) | 0.441 |
| **Strength testing^‡^** | | | | | | |
| Knee extension 1 RM (kg) | 44 | 23.1 (18.9) | *< 0.0001* | 44 | 15.3 (18.5) | *< 0.0001* |
| Maximum voluntary isometric contraction (Nm) | 46 | 11.8 (12.7) | *< 0.0001* | 45 | 6.74 (14.5) | *0.003* |
| Power (W) | 46 | 29.4 (40.7) | *< 0.0001* | 44 | 14.3 (35.7) | *0.011* |
| Relative strength (kg/kg)^§^ | 44 | 19.7 (19.0) | *< 0.0001* | 44 | 14.5 (17.9) | *< 0.0001* |
| **Immunohistochemistry** | | | | | | |
| Type I fiber CSA (µm^2^) | 30 | 7.64 (31.6) | 0.195 | 30 | 1.30 (23.1) | 0.760 |
| Type II fiber CSA (µm^2^) | 30 | 18.5 (31.5) | *0.003* | 30 | 14.5 (29.7) | *0.012* |
| Type I fiber frequency (%) | 30 | -6.06 (11.5) | *0.007* | 30 | 2.50 (12.4) | 0.278 |
| Type IIa fiber frequency (%) | 30 | 12.9 (12.6) | *< 0.0001* | 30 | 7.48 (17.0) | *0.023* |
| Type IIx/IIax fiber frequency (%) | 30 | -7.49 (14.4) | *0.008* | 30 | -10.1 (15.0) | *0.001* |
| Type I associated satellite cells/  Type I fiber | 30 | 16.1 (48.0) | 0.077 | 30 | 39.4 (56.0) | *< 0.001* |
| Type II associated satellite cells/  Type II fiber | 30 | 33.0 (79.1) | *0.030* | 30 | 32.1 (47.8) | *0.001* |
| CD11b/CD206 macrophages/fiber | 30 | 53.6 (66.3) | *< 0.001* | 30 | 57.3 (48.6) | *< 0.0001* |
| All CD11b macrophages/fiber | 30 | 50.9 (64.6) | *< 0.001* | 30 | 51.0 (50.6) | *< 0.0001* |

SD = standard deviation, ^†^Single sample t-test, ^‡^Baseline strength testing was performed at week 2 of PRT,

RM = repetition maximum, ^§^kg knee extension/kg thigh muscle mass

Table 2, continued. Within-group responses to PRT

|  | **PLACEBO** | | | **METFORMIN** | | |
| --- | --- | --- | --- | --- | --- | --- |
| **OUTCOME MEASURE** | **N** | **% change**  Mean (SD) | ^†^**p =** | **N** | **% change**  Mean (SD) | ^†^**p =** |
| **Western blotting** | | | | | | |
| phospho-AMPK/total AMPK (AU) | 14 | 1.63 (33.0) | 0.856 | 15 | 21.3 (25.5) | *0.006* |
| phospho-ACC1/total ACC1 (AU) | 15 | 6.24 (30.5) | 0.442 | 15 | 42.2 (53.9) | *0.009* |
| phospho-RPS6/total RPS6 (AU) | 15 | 73.1 (87.5) | *0.006* | 15 | 29.9 (31.7) | *0.003* |
| **Thigh muscle CT^¶^** | | | | | | |
| Low density muscle area,  0-34 HU (cm^2^) | 40 | -6.74 (11.6) | *< 0.001* | 37 | -3.86 (10.6) | *0.033* |
| Normal density muscle area,  35-100 HU (cm^2^) | 40 | 10.5 (7.01) | *< 0.0001* | 37 | 4.16 (9.11) | *0.009* |
| Thigh muscle area, 0-100 HU (cm^2^) | 40 | 6.43 (5.45) | *< 0.0001* | 37 | 2.27 (6.91) | 0.054 |
| Thigh muscle density (HU) | 40 | 4.13 (3.27) | *< 0.0001* | 37 | 2.49 (2.75) | *< 0.0001* |

SD = standard deviation, ^†^Single sample t-test, AU = arbitrary units, CT = computed tomography,

^¶^CT analyses are based on the mean of both legs, HU = Hounsfeild units
