# Supplementary material for: Metformin blunts muscle hypertrophy in response to progressive resistance exercise training in older adults: A randomized, double‐blind, placebo‐controlled, multicenter trial: The MASTERS trial
Source: Aging Cell. 2019 Sep 26;18(6):e13039. doi: 10.1111/acel.13039 (PMC6826125; doi:10.1111/acel.13039)
Supplement: Supplementary file 3 [file ACEL-18-e13039-s003.docx]

**Appendix S3. Changes in body weight, diet, and glucose metabolism in metformin versus placebo**

Eighty eight participants completed ≥3 days of diet records at both baseline and post intervention. Neither total calorie intake/day nor protein intake/day was affected by metformin. PRT led to a mean decrease in fasting glucose, but metformin had no effect on changes in fasting glucose. PRT also led to a significant increase in insulin sensitivity (SI) which was not affected by metformin treatment. Our study exclusion criteria included diagnosis of type 2 diabetes using HbA1c > 6.5 or fasting glucose > 126 mg/dL, but the exclusion criteria did not include 2 hour OGTT glucose > 200 mg/dL. Therefore, impaired fasting glucose (100-125 mg/dL) at baseline occurred in 20 participants who were randomized to placebo and 14 participants who were randomized to metformin. At baseline, impaired glucose tolerance (2 hour OGTT glucose 140-199 mg/dL) was observed in 13 placebo subjects and 8 metformin subjects. Type 2 diabetes, defined as 2 hour OGTT glucose > 200 mg/dL, occurred in 1 placebo subject and 2 metformin subjects. At both baseline and following 14 weeks PRT, the Matsuda method was used to calculate the insulin sensitivity index (ISI).

Table 1. Change in body weight, diet, and glucose metabolism

|  | **PLACEBO** | | | | **METFORMIN** | | | |  |
| --- | --- | --- | --- | --- | --- | --- | --- | --- | --- |
| **OUTCOME MEASURE** | **N** | **Baseline** | **14 week PRT** | **% change**  mean (SD) | **N** | **Baseline** | **14 week PRT** | **% Change**  mean (SD) | **p =** |
| **Body weight (kg)**  mean (SD) | 48 | 73.0  (13.4) | 71.8  (13.2) | -1.63  (3.75) | 46 | 79.3  (12.7) | 77.5  (12.7) | -2.36  (3.16) | 0.307 |
| **Diet** | | | | | | | | | |
| Food intake (kcal/day)  mean (SD) | 45 | 1768  (604) | 1676  (400) | -0.56  (22.9) | 43 | 1873  (548) | 1920  (616) | 5.0  (27.9) | 0.180 |
| Protein intake (g/day)  mean (SD) | 45 | 73.0  (19.5) | 69.5  (18.9) | -0.32  (32.3) | 43 | 77.6  (25.1) | 74.3  (26.3) | -1.1  (30.2) | 0.943 |
| **Glucose metabolism** | | | | | | | | | |
| Fasting glucose (mg/dL)  median (IQR) | 48 | 96.0  (89.3-102) | 92.8  (86.8-98.) | -3.35  (7.46) | 46 | 95.0  (86.5-102.8) | 92.8  (86.6-98.4) | -1.59  (7.99) | 0.272 |
| Insulin Sensitivity Index (Matsuda)  median (IQR) | 48 | 4.9  (3.1-6.4) | 5.23  (4.22-7.78) | 36.3  (33.6) | 46 | 4.1  (2.4-6.2) | 5.1  (3.2-7.3) | 43.3  (34.9) | 0.325 |

PRT = progressive resistance training, SD = standard deviation, IQR = interquartile range
