# Supplementary material for: Metformin blunts muscle hypertrophy in response to progressive resistance exercise training in older adults: A randomized, double‐blind, placebo‐controlled, multicenter trial: The MASTERS trial
Source: Aging Cell. 2019 Sep 26;18(6):e13039. doi: 10.1111/acel.13039 (PMC6826125; doi:10.1111/acel.13039)
Supplement: Supplementary file 4 [file ACEL-18-e13039-s004.docx]

**Appendix S4. Correlations between measures of change in muscle size**

Figure 1. Correlations between measurements of change in muscle size following PRT. Correlations included all subjects for whom we had all three measures: type II fiber cross-sectional area (CSA) (immunohistochemistry), bilateral thigh muscle mass (by DXA), and average thigh muscle area (by CT). N = 50. (a) There is a trend toward a correlation between change in type II fiber CSA and change in bilateral thigh muscle mass (R^2^ = 0.071, p = 0.058). (b) Change in type II fiber CSA is positively correlated to change in average thigh muscle area (R^2^ = 0.116, p = 0.014). (c) Change in bilateral thigh muscle mass is highly correlated to change in thigh muscle area (R^2^ = 0.325, p < 0.0001). In multivariate modeling, treatment group did not affect these relationships.
