# Supplementary material for: Metformin blunts muscle hypertrophy in response to progressive resistance exercise training in older adults: A randomized, double‐blind, placebo‐controlled, multicenter trial: The MASTERS trial
Source: Aging Cell. 2019 Sep 26;18(6):e13039. doi: 10.1111/acel.13039 (PMC6826125; doi:10.1111/acel.13039)
Supplement: Supplementary file 5 [file ACEL-18-e13039-s005.docx]

**Appendix S5. Additional Methods**

**Study Participants**

We sought to recruit 120 subjects, with an estimated 20% attrition, for a total of 100 subjects completing the trial. At both UAB and UK, participants were identified through responses to flyers, poster boards, newspaper advertisements, and web sites and volunteer databases including ResearchMatch.

Potential participants were excluded from the study if they met any of the following criteria: obese (BMI > 30); serum creatinine >1.4 because of risk of lactic acidosis with metformin; diabetes mellitus (HbA1c > 6.5 or fasting glucose > 126 mg/dL); uncontrolled hypertension; current angina pectoris or symptoms of myocardial ischemia or congestive heart failure; history (or ECG evidence) of previous myocardial infarction, history of congestive heart failure; chronic aspirin or NSAID use (unless it could be safely stopped prior to the biopsies), and any other use of an anticoagulant (e.g., coumadin) or history of bleeding; history of hypo- or hyper-coagulation disorders including subjects taking coumadin; lidocaine allergy (1% lidocaine is the local anesthetic used during the muscle biopsy procedure); history of alcoholism or liver disease; any end-stage disease and/or a life expectancy less than one year; neurological, musculoskeletal, or other disorder that would preclude them from completing resistance training and all performance tests; history of regular resistance training within the past year; any other medical condition that would interfere with testing or increase one's risk of complications during exercise, as judged by the study physicians; any other condition or events considered exclusionary by the PI and/or physicians.

**Randomization**

Following informed consent and establishment of eligibility, participants were randomized to receive either metformin or placebo. Randomization was stratified by site (UAB versus UK) and by functional status (low SPPB score (4-9) versus high SPPB score (10-12)) in permuted blocks of 4 to achieve balance between treatment groups. Randomization was performed by a statistician using SAS v9.2 PROC PLAN.

**Blinding**

No interim analyses on primary clinical outcomes related to metformin versus placebo assignment were performed. Research team members who had direct contact with study participants were blinded to group assignment until completion of the trial. However, research team members were able to view aggregate data for the purposes of monitoring procedures and measurements in order to ensure consistency between sites and consistent execution of the protocol over time. The placebo tablets were obtained from the Biomedical Research Institute of New Mexico VA Cooperative Studies Program Clinical Research Pharmacy Coordinating Center, and looked identical to the metformin tablets, thereby maintaining the double-blind study design. Tablets were dispensed by the UAB and UK research pharmacists, and given to participants by the study coordinators. Subjects were asked to return all leftover tablets to study coordinators for counting and disposal by the pharmacists.

**Insulin Sensitivity**

Subjects underwent oral glucose tolerance tests according to the World Health Organization standard protocol (75 g glucose, 2 h). Subjects arrived fasting and blood was drawn at baseline and 30, 60, 90 and 120 minutes after drinking 75 g of glucose. Plasma glucose was measured at each time point using the YSI Biochemistry Analyzer (YSI Incorporated, Yellow Springs, OH). Insulin was measured using the Meso Scale Discovery Human Insulin Kit (#K151BZC; MSD, Rockville, MD). The Matsuda insulin sensitivity index (ISI)^1^ was used to calculate insulin sensitivity, according to the formula: ISI (Matsuda) = 1000/(√G_0_*I_0_*G_mean_*I_mean_),

where

G_0_ = fasting plasma glucose concentration (mg/dl),

I_0_ = fasting plasma insulin concentration (mIU/l),

G_mean_ = mean plasma glucose concentration during OGTT (mg/dl), and

I_mean_ = mean plasma insulin concentration during OGTT (mU/l).

**Strength Testing**

Subjects underwent strength testing 5 times throughout the protocol: a familiarization session during pre-treatment (week 0); after 2 weeks of metformin or placebo (week 2); after 2 weeks of resistance training (week 4); at the midpoint of the resistance training program (week 9); and during the last week of PRT (week 16). Familiarization sessions were designed to accustom the subjects to the different exercises using proper technique and to explain 1RM testing. Week 4 (after 2 weeks of resistance training) strength testing was used as the baseline 1RM measurement, in order to account for the initial neuromuscular adaptations occurring at the beginning of the resistance training program.

Voluntary, dynamic strength was evaluated with the repetition maximum (1RM) using our well-established methods that were standardized across sites ^2,3^. Subjects performed two warm-up sets, with the first at an estimated 40–50% of 1RM weight and the second at an estimated 70–80% of 1RM weight. Single repetition trials, separated by 1–2 minutes of rest, were performed with increasing resistance until subjects experienced two failed attempts at a given weight. The largest load lifted with good form and range of motion was recorded as the 1RM. Verbal encouragement was given throughout all lifts, and 1RM testing was performed on high intensity workout days. Maximal voluntary isometric knee extension strength was tested using our established methods on a Biodex 4 dynamometer with a knee angle of 60°, using the maximal effort obtained over four 4-second trials ^4^.

**Computed Tomography (CT)**

The dominant leg was measured and marked on each participant so that images were obtained at the mid-thigh defined as the mid-point between the inguinal crease and proximal border of the patella. All acceptable images were free of artifact and contained the entire thigh in the field of view. In order to ensure that both baseline and week 16 images were taken at the same level of the mid-thigh, femur cross-sectional area was assessed using the polygon tool in Image J. Week 16 experimental data were excluded due to technical error when the difference between Baseline and week 16 femur CSA was greater than 5%. If baseline and week 16 were acceptable for analysis, the muscle area was outlined using the polygon tool in Image J. Hounsfield Unit (HU) threshold ranges were used to quantify the area of low density muscle (0 to 34 HU), and normal density muscle (35-100 HU). Mean HU for the total muscle area was also included in the analysis.

**Human myogenic progenitor cell isolation**

Muscle biopsies were disassociated using Collagenase II (800 U/ml) and Dispase (2.4 U/ml) in Ham’s F-10 plus 10% HS and 1% penicillin/streptomycin for 1 hour at 37°C with gentle agitation. The cell suspension was then passed through three cell strainers (100 μm, 70 μm, and 40 μm). Following centrifugation at 300 x g for 5 minutes at 4°C, the cell pellet was re-suspended in RBC lysis buffer and incubated at RT for 10 minutes. RBC lysis buffer was neutralized with wash media. Cells were pelleted by centrifugation at 300 x g for 5 minutes at 4°C and re-suspended in 500 μL of wash media. FC block TruStain (1μg/mL) (BioLegend) was added prior to antibody incubations for 15 mins on ice. Satellite cells were sorted using FACS for surface markers including anti-human CD31-FITC (1:100, Biolegend), CD34-FITC (1:100, Biolegend), CD45-FITC (1:100, Biolegend), and CD56-APC (1:20, Biolegend). Myogenic progenitor cells were identified as CD31-/CD34-/CD45-/CD56+.

**Immunohistochemistry**

Histological mounts were made in the biopsy procedure room immediately following the biopsy. Muscle pieces (~0.5-0.75 cm in length) were arranged with fibers in parallel and mounted perpendicular to the cork in mounting medium (1 part tragacanth powder, 1.5 parts O.C.T). The mount was then placed muscle-side down into isopentane chilled to the temperature of liquid nitrogen. For slides, 7 µm sections were cut in a cryostat (ThermoFisher) and allowed to air dry for at least 1 hour. Antibodies and dilutions are given in Table 1. To ensure that only the largest and highest quality mounts were analyzed, we eliminated sections that had fewer than 300 cross-sectional fibers, freeze damage, or were folded or torn. This left us with 31 complete sets from the placebo group and 33 complete sets from the metformin group. Out of these, we used 30/group with the largest number of cross-sectional fibers. Torn and folded portions of tissue sections were excluded from all analyses. For CSA and fiber type quantification, the mean number of fibers analyzed was 1105 (SD 476, range 408-2874).

*Fiber type-specific CSA:* To identify type I fibers, type IIa fibers, type IIx fibers, and fiber borders (laminin), unfixed slides were incubated overnight at 4°C in antibodies against MyHC I, MyHC IIax, MyHC IIx, and laminin. The next morning, slides were washed in PBS and then incubated in fluorophore conjugated secondary antibodies 1 hour at RT.

*Fiber type-specific Pax7:* Slides were fixed in ice-cold acetone for 5 minutes and then endogenous peroxidases were blocked with 3% H_2_O_2_ for 10 minutes. Afterwards, slides were blocked in 2.5% normal horse serum (NHS; Vector Labs) for 1 hour at RT. Slides were in incubated overnight at 4°C in Pax7, MyHC I, and laminin diluted in 2.5% NHS. The next morning, slides were incubated in GtαMs IgG1 biotin for 75 minutes at RT. Next, slides were incubated in streptavidin horseradish peroxidase (1:500; ThermoFisher A-11034, 2.5 µg/µl stock), GtαRb AF488, and GtαMs AF647 for 75 minutes at RT. Slides were then incubated in TSA AF594 for 15 minutes at RT and then incubated in DAPI for 10 minutes (Invitrogen).

*M1 and M2 macrophages:* Macrophage IHC was performed according to our validated protocol^5^. Slides were fixed in ice-cold acetone for 3 minutes. Endogenous peroxidases were quenched in 3% H_2_O_2_ for 8 minutes at RT, and then streptavidin/biotin blocking was performed according to manufacturer recommendations (Vector Laboratories SP-2002). Slides were next blocked overnight at 4°C in 2.5% NHS followed by incubation in primary antibody for CD11b for 24 hours at 4°C. Next, sections were incubated in GtαMs IgG1 biotin for 90 minutes at RT, followed by incubation streptavidin horseradish peroxidase (1:500) for 1 hour at RT. Slides were incubated in TSA AF488 for 20 minutes at RT. Streptavidin/biotin blocking and 2.5% NHS blocking were repeated, and then slides were incubated in primary antibody for CD206 overnight at 4°C. RbαGt biotin (Vector Laboratories MP-5402) was added for 90 minutes at RT. Slides were incubated in SA AF594 for 60 minutes at RT and then DAPI for 10 minutes.

For all stains, 3 x 5-minute PBS washes were performed between steps. Following the completion of each staining protocol, slides were mounted with fluorescent mounting media (Vectashield, #H-1000; Vector Laboratories Inc., Burlingame, CA) and then imaged. Mosaic images were taken at either 10x (fiber type-specific CSA images) or 20x (Pax7 and macrophage images) magnification using a Zeiss upright microscope (AxioImager M1) equipped with Zen 2.3 acquisition software (Zeiss, Oberkochen, Germany). Fiber type and CSA images were analyzed using our validated software package, MyoVision^6^.

**Western blotting**

Antibodies and dilutions are given in Table 1. Snap-frozen muscle pieces and myotubes were homogenized in 10 volumes Cell Signaling Lysis Buffer (Millipore 43-040) with protease and phosphatase inhibitors and then sonicated for 3 x 10 second bursts. Samples were then spun down at 10,000 x g for 15 minutes at 4°C. The supernatant was then collected and a Bradford protein assay was performed. 30 μg of protein was resolved into 4-15% Criterion TGX Precast Midi Protein Gels (Bio-Rad), and then transferred onto nitrocellulose membranes (Millipore Sigma). After blocking in a 50:50 mixture of TBS Odyssey Blocking Buffer (LI-COR Biosciences) and TBS plus 0.1 % Tween-20 (TBS-T) for 1 h at room temperature, membranes were incubated overnight at 4°C in blocking buffer and TBS-T with the respective phospho- primary antibody, including phospho-AMPK, phospho-ACC, phospho-RPS6, or phospho-p70S6K1. The next morning, membranes were incubated in near-infrared conjugated secondary antibodies in TBS-T for 1 hour and imaged using the LI-COR Odyssey Infrared Imaging System (LI-COR Biosciences). Blots were then stripped using Restore Fluorescent Western Blot Stripping Buffer (Thermo Scientific) and then blocked again. Membranes were then incubated overnight at 4°C in blocking buffer and TBS-T with the respective primary antibody, including total AMPK, ACC2, RPS6, or p70S6K1. Lastly, membranes were incubated in near-infrared conjugated secondary antibodies in TBS-T for 1 hour and imaged using the LI-COR Odyssey Infrared Imaging System. Total protein was stained using REVERT Total Protein Stain (LI-COR Biosciences). Blots were quantified using the LI-COR Image Studio Software.

**Table 1. Antibodies**

| **Primary Antibody** | **Dilution** | **Catalog number, Vendor** | **Secondary Antibody** | **Dilution** | **Catalog number, Vendor** | **Fluorescent amplification** |
| --- | --- | --- | --- | --- | --- | --- |
| CD11b | 1:100 | MON1019,  Cell Sciences | GtαMs IgG1 biotin | 1:1000 | 115-065-205; Jackson Immuno | TSA AF488 |
| CD206 | 1:200 | AF2534,  R & D Systems | RbαGt IgG biotin | 1:500 | BA-5000,  Vector Labs | SA AF594 |
| Laminin | 1:100 | L9393,  Sigma Aldrich | GtαRb  AMCA | 1:100 | CI-1000,  Vector Labs | None |
| Pax7 | 1:100 | DSHB | GtαMs biotin | 1:1000 | 115-065-205; Jackson Immuno | TSA AF594 |
| Type I fibers | 1:100 | BA.D5,  DSHB | GtαMs IgG2b AF647 | 1:250 | A21242,  Invitrogen | None |
| Type IIax fibers | neat | SC.71 IgG1, DSHB | GtαMs IgG1 AF488 | 1:500 | A21121, Invitrogen | None |
| Type IIx fibers | neat | 6H1 IgM,  DSHB | GtαMs IgM AF555 | 1:500 | A21426, Invitrogen | None |
| Phospho-AMPK Thr172 | 1:1000 | 07-681, Millipore | IRDye® 800CW GtαRb | 1:10,000 | 926-32211, LI-COR Biosciences | None |
| AMPK α1 | 1:1000 | 07-350, Millipore | IRDye® 680RD GtαRb | 1:10,000 | 926-68071, LI-COR Biosciences | None |
| phospho-ACC Ser79 | 1:1000 | 07-303, Millipore | IRDye® 800CW GtαRb | 1:10,000 | 926-32211, LI-COR Biosciences | None |
| ACC1 | 1:1000 | MABS830, Millipore | IRDye® 680RD GtαMs | 1:10,000 | 926-68070, LI-COR Biosciences | None |
| phospho-RPS6  Ser240/244 | 1:1000 | 5364S, Cell Signaling | IRDye® 800CW GtαRb | 1:10,000 | 926-32211, LI-COR Biosciences | None |
| RPS6 | 1:1000 | 2217S, Cell Signaling | IRDye® 680RD GtαRb | 1:10,000 | 926-68071, LI-COR Biosciences | None |
| phospho-  p70S6K1  Thr389 | 1:1000 | 97596S, Cell  Signaling | IRDye® 800CW GtαRb | 1:10,000 | 926-32211, LI-COR Biosciences | None |
| p70S6K1 | 1:1000 | 2708S, Cell  Signaling | IRDye® 680RD GtαRb | 1:10,000 | 926-68071, LI-COR Biosciences | None |

**Statistics and Data Management**

For data quality assurance, handwritten notes were entered by two different investigators into duplicate columns into RedCap project. Duplicate data entry was performed for 3685 fields, representing 25 variables from 55 subjects from both sites and all time points. We identified 16 discrepancies, which represent data entry errors in 0.43% of fields. A decision was made to discontinue the quality assurance process given the proportion of discrepancies was smaller than 5%, which was the threshold for the process to continue.

*Power Calculations:* The trial was designed to test the primary hypothesis that metformin would augment fiber hypertrophy in response to resistance exercise training in elderly people. For power calculations, we used data from two of our prior resistance exercise trials in older adults (25 men, 25 women, aged 60–80 years) ^7,8^, in which baseline type II fiber CSA means were 4095 (± 1213 SD) μm^2^ in men and 2458 (± 690 SD) μm^2^ in women. Based on previous work, we expected that 14 weeks of PRT alone would be associated with an approximate 20% increase in type II fiber CSA in both men and women. We further predicted that adding metformin would yield an additional 25% increase in fiber CSA (above the 20% increase due to PRT alone). With a 20% increase in PRT alone and an additional 25% increase with metformin, the mean difference between treatment groups would be at least 730 μm^2^, assuming a pooled standard deviation of 950 μm^2^. Thus, the effect size is expected to be at least 0.76. A two-sample t-test would have at least 80% power to detect effect sizes of 0.57 and larger with an overall sample of 100 participants.

**REFERENCES**

1 Matsuda, M. & DeFronzo, R. A. Insulin sensitivity indices obtained from oral glucose tolerance testing: comparison with the euglycemic insulin clamp. *Diabetes Care* **22**, 1462-1470 (1999).

2 Dennis, R. A. *et al.* Muscle expression of genes associated with inflammation, growth, and remodeling is strongly correlated in older adults with resistance training outcomes. *Physiol Genomics* **38**, 169-175, doi:10.1152/physiolgenomics.00056.2009 (2009).

3 Petrella, J. K., Kim, J. S., Tuggle, S. C., Hall, S. R. & Bamman, M. M. Age differences in knee extension power, contractile velocity, and fatigability. *Journal of applied physiology* **98**, 211-220, doi:10.1152/japplphysiol.00294.2004 (2005).

4 Srikuea, R. *et al.* Association of fibromyalgia with altered skeletal muscle characteristics which may contribute to postexertional fatigue in postmenopausal women. *Arthritis Rheum* **65**, 519-528, doi:10.1002/art.37763 (2013).

5 Kosmac, K. *et al.* Immunohistochemical Identification of Human Skeletal Muscle Macrophages. *Bio Protoc* **8**, doi:10.21769/BioProtoc.2883 (2018).

6 Wen, Y. *et al.* MyoVision: software for automated high-content analysis of skeletal muscle immunohistochemistry. *Journal of applied physiology* **124**, 40-51, doi:10.1152/japplphysiol.00762.2017 (2018).

7 Kosek, D. J., Kim, J. S., Petrella, J. K., Cross, J. M. & Bamman, M. M. Efficacy of 3 days/wk resistance training on myofiber hypertrophy and myogenic mechanisms in young vs. older adults. *Journal of applied physiology* **101**, 531-544, doi:10.1152/japplphysiol.01474.2005 (2006).

8 Stec, M. J. *et al.* Randomized, four-arm, dose-response clinical trial to optimize resistance exercise training for older adults with age-related muscle atrophy. *Exp Gerontol* **99**, 98-109, doi:10.1016/j.exger.2017.09.018 (2017).
